# Is functional fitness performance a useful predictor of risk of falls among community-dwelling older adults?

**DOI:** 10.1186/s13690-021-00608-1

**Published:** 2021-06-18

**Authors:** Hsin-Hung Ho, I-Yao Fang, Yi-Chien Yu, Yi-Ping Huang, I-Ling Kuo, Li-Ting Wang, Ming-Chueh Tsai, Shao-Hsi Chang, Ming-Chun Hsueh

**Affiliations:** 1 Department of Geriatric Care, Mackay Junior College of Medicine, Nursing and Management, 92, Shengjing Road, Taipei, 112 Taiwan; 2 Physical Education Center, Southern Taiwan University of Science and Technology, 1, Nan-Tai Street, Yungkang Dist, Tainan, 710301 Taiwan; 3 Department of Physical Education, National Taiwan Normal University, 162, Heping East Road Section 1, Taipei, 106 Taiwan; 4 Graduate Institute of Sport, Leisure and Hospitality Management, National Taiwan Normal University, 129, Heping East Road Section 1, Taipei, 106 Taiwan; 5 Graduate Institute of Sport Pedagogy, University of Taipei, 101, Jhongcheng Road Section 2, Taipei, 111 Taiwan

**Keywords:** Elderly, Physical performance, Physical function, Frailty, Taiwan

## Abstract

**Background:**

Falls among older adults are a serious public health problem. Many studies indicate that positive functional fitness performance decreases the risk of falls. A limited amount of previous study has investigated the association between broad functional fitness and the fall risk. This study examines the associations between functional fitness and the risk of falling among community-dwelling older adults.

**Methods:**

Three waves of cross-sectional data were collected from 2017 to 2019 in Taipei City, Taiwan. Six hundred sixty-five participants aged ≥65 years were randomly recruited from 12 districts of Taipei. Eight functional fitness tests (i.e., back scratch, chair-sit and-reach, 8-ft up-and-go, 30-s sit-to-stand, 30-s arm curl, 30-s single-leg stance, 2-min step, and hand grip strength tests) were performed to record the physical performance of older subjects. A Chinese version of the fall-risk questionnaire (FRQ) was used to calculate the fall risk scores. Linear regression and logistic regression were utilized to estimate the relationships of each functional fitness and fall risk.

**Result:**

The results showed that 37.45% of older adults had a high risk of falling. It was found for each functional fitness that performance was linearly associated with the risk of falling. Moreover, older adults with low-performance levels in all functional fitness except back-scratching were more likely to have a higher risk of falling.

**Conclusions:**

Our study indicated that functional fitness performance appears to provide valid predictive guidance for reducing the risk of falling among the older population.

**Supplementary Information:**

The online version contains supplementary material available at 10.1186/s13690-021-00608-1.

## Introduction

According to epidemiological studies, a fall is a public concern which results older person coming to rest inadvertently on the ground or floor [1]. Globally, 45% of people in long-term care experience falls, and 40% of them experience diverse falls per year [1–3]. Falls and fall-associated injuries (e.g., fractures) are responsible for high levels of morbidity, immobility, and mortality among older people [4] and might resulted losing independence, requiring hospitalization and even dying [5]. There is therefore an urgent need to identify the underlying correlations associated with the risk of falling among older adults.

There is growing interest in the adaptation of physical functions, such as improved mobility, balance and muscle strength, all of which are important in preventing falls among older adults [6, 7]. The functional fitness is a comprehensive instrument for assessing older adults’ physical functions [8]. The functional fitness is a widely-used measurement of independence, health and life quality for adults in later life [9]. Rikli & Jones [10] validated a functional fitness battery for community-dwelling older adults. Aspects of functional fitness such as muscle strength, walking speed, flexibility, cardio-endurance and balance, were found to be important to the fall prevention of older persons [11]. Although the associations between functional fitness and the fall risk have been well documented [7], specific limitations with comprehensive functional fitness and fall risk need to be confirmed.

Previous studies have not consistently illustrated the association between each functional fitness index and fall risk among older adults [7, 12]. For example, Zhao and Chung [7] reported that older adults who risked falling had lower capacity for time-up-and-go tests (morbidity), arm curls (upper-muscle strength) and 2-min steps (cardio-endurance) compared with those who were not at risk of falling. However, no differences were observed in lower-muscle strength and flexibility. Smee et al. [12] have shown that upper and lower-body strength, balance and endurance, but not upper-body flexibility, are associated with the risk of falling. Moreover, one study using a short physical-performance battery found only that static balance was associated with the physiological risk of falling [6].

Furthermore, fall-risk evidence that is based on the assessment of a single physical function (e.g. gait speed alone) might lack comparability for public health guidance [13–15]. The current evidence to determine the discriminant and predictive validity for fall-risk of functional fitness in community-dwelling older adults is unclear [6]. Even though threshold values are well reported in literature, detecting the optimal cut-off value at each functional fitness level to prevent falls in older adults remains debatable.To apply suitable functional fitness examinations and guide customized exercise interventions to minimize fall risk in community-dwelling older adults, further studies are needed to determine population-based cut-off values and the generalization of findings. To fill these gaps in the literature, this study aimed to explore the dose-response association between each functional fitness test and fall risk.

## Methods

### Participants

This study involved three waves cross-sectional data collected from 2017 to 2019 in Taipei city, Taiwan. Eight hundred eighty-six potential participants were randomly recruited from 12 neighborhoods: interested individuals contacted the registered nurse or neighborhood representatives. The participants were people living independently in the community who were aged 65 and above. In the recruitment process, the trained nurse screened the participant’s health evaluations. Therefore, all participants were community-dwelling volunteers who passed comprehensive health and functional screening evaluations, and were free of major chronic conditions and cognitive and functional impairment at the time of attending this study. Furthermore, participants were asked questions from the fall-risk questionnaire and underwent functional fitness tests organized by a team of trained research assistants.

**Fig. 1:**
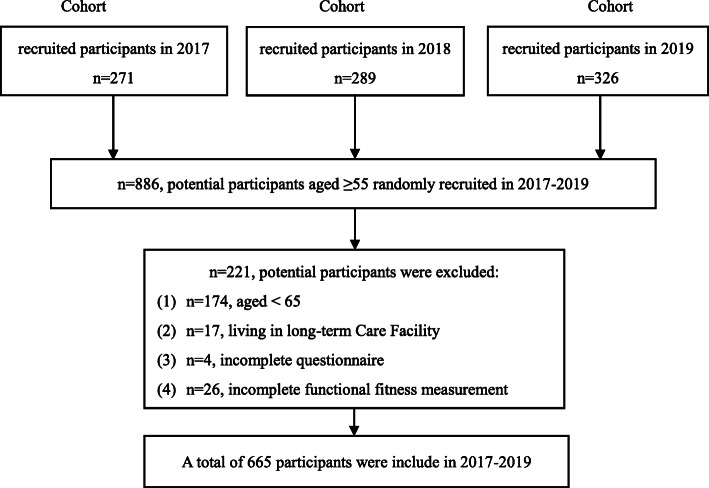
Flowchart of participant selection process based on inclusion criteria

The exclusion criteria included: age below 65y (*n* = 174); residence in long-term care facility (*n* = 17); incomplete or missing data in fall-risk questionnaire (*n* = 4); and incomplete functional fitness tests (*n* = 26). Hence, 221 potential participants were excluded. After data cleaning, 665 completed data records that were valid for analysis were obtained. A flow diagram of the study recruitment is presented in Fig. 1. All participants were informed about the nature and purposes of the study, and written informed consent was obtained from each subject. Ethical approval was received from the Research Ethics Committee of the National Taiwan Normal University (REC number: 201703HM010-201805HM002-201905HM042).

### Measures

#### Functional fitness performance

Senior Fitness Test was used to measure older adult’s functional fitness performance [10]. Previous study had extensively described the validity and reliability of functional fitness [8]. There are eight functional fitness items assessing the five physical functional dimensions:

1. A 30-s sit-to-stand test, to evaluate the lower limbs’ muscle strength. Participants were asked to rise from their chair to a full standing position and then return to a seated position, to see how many stands could be completed in 30 s with the arms folded across the chest.
2. Arm-curl test. Participants held a dumbbell in their usual hand, women holding a weight of 5 lbs., men holding 8 lbs. The number of biceps curls that could be completed in 30 s was recorded.
3. Single-leg stance, to assess static balance. The participants stood on one foot until they lost their balance. Each participant was asked to perform this twice, and the longest performance was recorded.
4. 8-ft up-and-go test, to assess agility and dynamic balance. Participants were asked to rise from their chair, pace for a distance of eight feet (2.44 m), then go around a cone and return to their chair. Participants were asked to perform this twice, and their fastest time was recorded.
5. Back-scratch test, to assess upper-body flexibility. Participants were asked to stand and place their hands behind their back. The distance that their hands overlapped behind them was measured.
6. Chair sit-and-reach test to assess lower body flexibility. Participants were asked to sit on a chair keeping one leg straight out, to stretch their hands as far as possible towards their toes, and then to hold this position for 2 seconds.
7. 2-min step test, to assess aerobic endurance. Participants were asked to raise their knee to a prescribed height for as many times as possible within 2 minutes. The total number of steps was recorded.
8. The hand-grip strength test was also added to measure upper strength, using a grip dynamometer measuring in kilograms (TTM-YD; Accuratus, Taiwan). Before the test, the grip device was adjusted to fit each participant’s hand. Participants were instructed to stand and hold the device in one hand keeping their arms vertical and away from their bodies. The participants were then asked to squeeze the grip dynamometer using maximum force. Each participant was asked to perform this action twice with a one-minute interval between attempts. Their best performance was recorded.

The arm-curl test and the hand-grip strength test are recognized as valid and reliable ways of assessing upper-limb muscle strength in older adults [10].

#### Fall risk test

The fall-risk of all participants was assessed using the Chinese version of the fall-risk questionnaire (FRQ) [16]. This questionnaire was designed to identify whether participants had experienced a fall during the previous 12 months. Detailed information regarding the consisted of the 12-item questions has been presented elsewhere [16]. Each statement was to be answered by Yes or No, with a maximum score of 14 possible points. A high fall-risk of the participants was determined at ≥4 scores. The Cronbach’s alpha value of the Chinese version of the FRQ scale was 0.69.

### Covariates

The covariates included demographic variables: gender, age (65–74, ≥75), functional fitness item, and body-mass index (BMI, which was self-reported and calculated using height and weight), which was categorized into underweight (< 18 kg/m^2^), normal (18.5–23.9 kg/m^2^), overweight (24.0–26.9 kg/m^2^), and obese (≥27 kg/m^2^) according to Asian cut-off points [17].

### Statistical analyses

Descriptive statistics (mean and standard deviation) were calculated to determine the fall-risk categories to which participants belonged (in Table 1). An independent sample t-test and chi-square test were performed to identify the mean and proportional of the demographic variables difference by fall-risk categories, separately. The independent sample t-test was also used to examine the difference in functional fitness of the fall-risk groups. Partial Spearman’s rank correlation coefficients were conducted to examine the difference between the category and continuous variable of functional fitness and fall-risk after adjusting the covariates (age and BMI). Moreover, multiple linear regression and logistic regression were used to analyze the associations between functional fitness and the fall-risk adjustment of the covariates, separately. The level of confidence was set at 95% and the *p*-value was set at < 0.05. Data analysis was performed using IBM SPSS Statistics version 23.0 (SPSS Inc., Chicago, IL, USA).

**Table 1:** Characteristics of participants

Variable	Total sample	Low-risk (***n*** = 416)		High-risk (***n*** = 249)		***t***	***p-***value
	n, %, or *M* (±SD)	n, %; or *M* (±SD)		n, %; or *M* (±SD)			
Age	73.6 (±6.4)	72.8	(6.11)	74.9	(6.70)	−4.28	**< 0.001**
Gender	*n* = 524	1.78	(0.41)	1.80	(0.40)	−0.35	0.73
Older men	*n* = 141, 21.2%	*n* = 90	63.8%	*n* = 51	36.2%		
Older women	*n* = 524, 78.8%	*n* = 326	62.2%	*n* = 198	37.8%		
BMI (kg/ m^2^)	23.8 (±3.7)	23.65	(3.59)	24.11	(3.92)	−1.55	0.12
Underweight	*n* = 45; 6.8%	*n* = 29; 7.0%		*n* = 16; 6.4%			0.19
Normal	*n* = 316; 47.5%	*n* = 205; 49.3%		*n* = 111; 44.6%			
Overweight	*n* = 172; 25.9%	*n* = 110; 26.4%		*n* = 62; 24.9%			
Obese	*n* = 132; 19.8%	*n* = 72; 17.3%		*n* = 60; 24.1%			
Sit-to-stand (times)	18.68 (5.58)	19.66	(5.33)	17.04	(5.61)	6.01	**< 0.001**
Arm curl (times)	17.29 (4.41)	17.94	(4.35)	16.22	(4.30)	4.95	**< 0.001**
Single-leg stance (sec)	18.27 (11.04)	19.98	(10.72)	15.42	(10.98)	5.26	**< 0.001**
8-ft up-and-go (sec)	6.41 (1.91)	5.97	(1.45)	7.14	(2.32)	−8.02	**< 0.001**
Back scratch (cm)	−2.46 (12.09)	−2.08	(12.02)	−3.08	(12.20)	1.03	0.3
Chair sit-and-reach (cm)	4.60 (11.56)	5.88	(10.64)	2.48	(12.68)	3.70	**< 0.001**
2-min step (times)	95.07 (21.03)	98.18	(18.46)	89.89	(23.88)	5.01	**< 0.001**
Hand Grip Strength (kg)	24.35 (6.86)	25.44	(7.19)	22.51	(5.84)	5.44	**< 0.001**

Abbreviations: *n* number, *BMI* body mass index, *M* mean *SD* = standard deviation. *p* < 0.05

## Results

### Participants description

Table 1 shows the sociodemographic characteristics by status for fall-risk among older adults. The participants’ mean age was 73.6 (±6.4) years; 78.8% were older women; the mean (±SD) of BMI was 23.8 (±3.7). In functional fitness performance, the mean (±SD) for sit-to-stand was 18.68 s (±5.58 s). For the arm curl the mean was 17.29 (±4.4) repetitions, for the single-leg stance it was 18.27 s (±11.04 s), for the 8-ft up-and-go it was 6.41 s (±1.91 s), for the back-scratch it was − 2.46 (±12.09) cm, for the chair sit-and-reach it was 4.60 (±11.56) cm, for the 2-min step it was 95.07 (±21.03), and for the hand-grip strength it was 24.35 (±6.86) kg.

According to their FRQ score, participants were stratified into those with low fall-risk (< 4) and high fall-risk (≥4). 249 (37.45%) participants were categorized into the high fall-risk group. Among the subjects, 36.2% (*n* = 51) older men and 37.8% (*n* = 198) older women were classified as high fall-risk. Chi-square test analysis revealed proportional differences in BMI status (*p* = 0.193). The independent sample t-test revealed significant differences in functional fitness between low fall-risk (< 4) and high fall-risk (≥4) adults, including sit-to-stand (t = 6.0; *p* = <.001), arm curl (t = 4.9; *p* = <.001), single-leg stance (t = 5.2; *p* = <.001), 8-ft up-and-go (t = − 8.0; *p* = <.001), chair sit-and-reach (t = 3.7; *p* = <.001), 2-min step (t = 5.0; *p* = <.001), and hand-grip strength (t = 5.4; *p* = <.001). No difference was observed in back-scratch (t = 1.0; *p* = .301) between fall-risk groups.

### Partial correlations between functional fitness and fall-risk

As shown in Table 2, the associations between the functional fitness (sit-to-stand: *r* = −.21, *p* < .001; arm curl: *r* = −.20, *p* < .001; single-leg stance: *r* = −.17, *p* < .001; chair sit-and-reach: *r* = −.14, *p* < .001; 2-min step: *r* = −.16, *p* < .001; hand-grip strength: *r* = −.18, *p* < .001) were inversely associated with both continuous and categorical fall risk. In addition, the 8-ft up-and-go test (*r* = .33, *p* < .001) was positively associated with both continuous and categorical fall-risk. Only the back-scratch (*r* = −.02, *p* < .621) was not associated with fall-risk.

**Table 2:** Partial correlation coefficients between each functional fitness test and fall-risk

Variables	1	2	3	4	5	6	7	8	9	10
1. Sit-to-stand	1									
2. Arm curl	.52***	1								
3. Single-leg stance	.26***	.14***	1							
4. 8-ft up-and-go	−.52***	−.39***	−.28***	1						
5. Back-scratch	.08*	.04	.12**	−.14***	1					
6. Chair sit-and-reach	.31***	.22***	.15***	−.23***	.33***	1				
7. 2-min step	.47***	.34***	.25***	−.43***	.16***	.24***	1			
8. Hand-grip strength	.25***	.27***	.10*	−.23***	−.12**	−.08*	.16***	1		
9. Fall risk (categorical)	−.19***	−.18***	−.13**	.25***	.020	−.11**	−.15***	−.20***	1	
10. Fall risk (continuous)	−.21***	−.20***	−.17***	.33***	−.02	−.14***	−.16***	−.18***	.84***	1

*p < .05, **p < .01, ***p < .001

### Predictive factors of functional fitness with fall-risk

The multiple linear regression is shown in Table 3. The results in model 1, after adjustment for potential confounders, show each functional fitness index (sit-to-stand: β = −.21, [95% CI = −.14, −.29]; arm curl: β = −.20, [95% CI = −.12, −.27]; single-leg stance: β = −.20, [95% CI = −.11, −.28]; 8-ft up-and-go: β = .36, [95% CI = .44, .28]; chair sit-and-reach: β = −.14, [95% CI = −.06, −.21]; 2-min step: β = −.17, [95% CI = −.09, −.24]; and hand-grip strength: β = −.18, [95% CI = −.11, −.26]) were significantly associated with fall-risk (continuous). Only the back-scratch (β = −.02, [95% CI = .06, −.10]) was not correlated with fall-risk scores. Furthermore, we included all the significantly functional fitness variables with fall risk in a multiple linear regression (see Additional file 1). The results shown that 8-ft up-and-go, hand-grip strength and single-leg stance, were the major predictors for the risk of falling (data not shown).

**Table 3:** Bata values, odds ratios (ORs) and 95% confidence intervals (CI) for scoring in functional fitness and fall risk

Variable		Fall-risk score (continuous)		Fall-risk (categorical)	
		Model 1		Model 2	
		β (95% CI)	***p***-value	OR (95% CI)	***p***-value
**Sit-to-stand**	Continuous	−.21 (−.14, −.29)	**< 0.001**		
	Q1 High	–	**–**	1.00 (ref.)	
	Q2 Moderate	–	**–**	1.87 (1.25–2.79)	.002*
	Q3 Low	–	**–**	3.07 (2.06–4.56)	< 0.001*
**Arm curl**	Continuous	−.20 (−.12, −.27)	**< 0.001**		
	Q1 High	–	–	1.00 (ref.)	
	Q2 Moderate	–	–	1.66 (1.11–2.49)	.014*
	Q3 Low	–	–	2.47 (1.68–3.65)	<.001*
**Single-leg stance**	Continuous	−.20 (−.11, −.28)	**< 0.001**		
	Q1 High	–	–	1.00 (ref.)	
	Q2 Moderate	–	–	1.38 (0.91–2.09)	.14
	Q3 Low	–	–	2.14 (1.41–3.24)	<.001*
**8-ft up-and-go**	Continuous	.36 (.44, .28)	**< 0.001**		
	Q1 High	–	–	1.00 (ref.)	
	Q2 Moderate	–	–	1.85 (1.20–2.84)	.005*
	Q3 Low	–	–	4.01 (2.59–6.23)	<.001*
**Back-scratch**	Continuous	−.02 (.06, −.10)	.621		
	Q1 High	–	–	1.00 (ref.)	
	Q2 Moderate	–	–	0.91 (0.61–1.37)	.66
	Q3 Low	–	–	1.12 (0.74–1.69)	.59
**Chair sit-and-reach**	Continuous	−.14 (−.06, −.21)	**< 0.001**		
	Q1 High	–	–	1.00 (ref.)	
	Q2 Moderate	–	–	0.86 (0.57–1.29)	.46
	Q3 Low	–	–	1.68 (1.14–2.50)	.009*
**2-min step**	Continuous	−.17 (−.09, −.24)	**< 0.001**		
	Q1 High	–	–	1.00 (ref.)	
	Q2 Moderate	–	–	1.19 (0.80–1.77)	.40
	Q3 Low	–	–	1.87 (1.25–2.79)	.002*
**Hand Grip Strength**	Continuous	−.18 (−.11, −.26)	**< 0.001**		
	Q1 High	–	–	1.00 (ref.)	
	Q2 Moderate	–	–	1.76 (1.17–2.64)	.006*
	Q3 Low	–	–	2.58 (1.72–3.88)	<.001*

Abbreviations: *β (95% CI)* standardized regression coefficients and 95% confidence intervals, *OR* odds ratio, *CI* conference interval; Adjusted for age, gender, and Body Mass Index (BMI); * *p* < .05; Variable cut-off: Sit-to-stand (times) = Q1, 21 ~ 36; Q2, 17 ~ 20; Q3, 5 ~ 16. Arm curl (times) = Q1, 20 ~ 36; Q2, 17 ~ 19; Q3, 0 ~ 16. Single-leg stance (sec)=; Q1, 30.00; Q2, 10.01 ~ 29.99; Q3, 0.00 ~ 10.00. 8-ft up-and-go (sec) = Q1, 2.40 ~ 5.45; Q2, 5.46 ~ 6.60; Q3, 6.61 ~ 19.90. Back-scratch (cm) = Q1, 4.51 ~ 35.10; Q2, −5.41 ~ 4.50; Q3, − 50.00 ~ − 5.40. Chair sit-and-reach (cm) = Q1, 9.01 ~ 41.10; Q2, 1.01 ~ 9.00; Q3, − 45.00 ~ 1.00. 2-min step (times) = Q1, 105–165; Q2, 92–104; Q3, 0–91. Hand Grip Strength (kg) = Q1, 25.41 ~ 48.40; Q2, 21.01 ~ 25.40; Q3, 9.90 ~ 21.00

The results in model 2, the sit-to-stand test, show that compared with high levels of sit-to-stand performance, older adults with low (OR = 3.07, 95% CI = 2.06–4.56) and moderate (OR = 1.87, 95% CI = 1.25–2.79) sit-to-stand performance were more likely to have a higher fall-risk. With the arm-curl test, compared with high levels of arm curl capacity, older adults with low (OR = 2.47, 95% CI = 1.68–3.65) and moderate (OR = 1.66, 95% CI = 1.11–2.49) levels of arm curl capacity were more likely to have a higher fall-risk. For the single-leg stance test, compared with high levels of single-leg stance stances, older adults with low levels of single-leg stances (OR = 2.47, 95% CI = 1.68–3.65) were more likely to have a higher fall-risk. For the 8-ft up-and-go, compared with high-level groups, older adults with low (OR = 4.01, 95% CI = 2.59–6.23) and moderate (OR = 1.85, 95% CI = 1.20–2.84) mobility were more likely to have a higher fall-risk. For the chair sit-and-reach test, compared with the reference group, older adults with low levels of chair sit-and-reach (OR = 1.68, 95% CI = 1.14–2.50) were more likely to have a higher fall-risk. For the 2-min step test, compared with high levels of cardio endurance, older adults with low numbers of steps (OR = 1.87, 95% CI = 1.25–2.79) were more likely to have a higher fall-risk. For hand-grip strength, compared with robust groups, older adults with low (OR = 2.58, 95% CI = 1.72–3.88) and moderate (OR = 1.76, 95% CI = 1.17–2.64) levels of hand-grip strength were more likely to have a higher fall-risk. For the back-scratch, no index was found.

## Discussion

The aims of this study were (i) to determine whether each index of functional fitness capacity was associated with fall-risk in community-dwelling older adults; and (ii) to examine what cut-off point of functional fitness should be used when differentiating a high from a low risk of falling among community-living older adults. The main result of this study was that a dose-response relationship exists between each functional fitness index and fall-risk among older adults, except for upper flexibility. The most critical findings of the present study were that participants with low and moderate levels of sit-to-stand, arm curl, 8-ft up-and-go, and hand-grip strength were found to have a higher risk of falling, compared with relatively healthy and fit individuals. In addition, older adults with low-level performance in the single-leg stance, chair sit-and-reach, and 2-min step were associated with higher fall-risk, compared with older adults with higher levels of performance. Therefore, with respect to initiatives for the improving functional fitness in older adults, our findings may provide critical information for determining cut-off points for reducing fall-risk among older adults.

Previous studies demonstrated that older adults with poor mobility [7, 18, 19], poor static balance [6], poor lower-extremity strength [12], poor upper muscle strength, and lower cardio-endurance [7, 12] were at greater risk of falling. Our study supports this evidence. However, to date, there has been an ongoing discussion regarding what cut-off scores to recommend when differentiating a high risk from a low risk of falling among community-living older adults. To our knowledge, few previous studies have examined the cut-off points of the multicomponents of functional fitness in relation to fall-risk. For example, Shumway-Cook et al. [19] and Francisco et al. [20] suggest that the time-up-and-go test (not the 8-ft up-and-go) with a cut-off of 13.5 s is a sensitive and specific measure for identifying community-dwelling older adults and nursing home residents who are at risk of falls. In the same way, one way to prevent falls may be to ensure that adults in an institution aged 80 or above can achieve 6.5 repetitions in a 30-s chair sit-to-stand test [20]. However, these studies have not been adapted for public implementation, and involved a low number of participants from a non-randomized selection. In addition, previous evidence on the cut-off point for the 30-s single-leg stance test was unknown. Consequently, several cut-off points have been determined in this study and it could help determine optimal functional fitness performance levels for preventing falls among older populations.

Our study provides clear information regarding the cut-off points for mobility, static balance, muscle strength, flexibility and cardio-endurance performance in older adults at high risk of falling, which may strengthen surveillance systems that track fall-risk with a view to possible intervention. We therefore believe our study to be valuable for evaluating the cut-off thresholds of the association between multicomponents of functional fitness and fall risk in community-dwelling older adults. Specifically, we observed that an 8-ft up-and-go score of more than 6.6 s reflects a high fall-risk, and that this was the best predictor of fall-risk in terms of functional fitness. This finding was similar to a previous study based on the 8-ft walking test [7, 21]. The study by Vainshelboim et al. [21] reported that an 8-ft up-and-go score greater than or equal to 6.9 s was found to be associated with hospitalization and mortality. The 8-ft up-and-go test is a modified version of the time-up-and-go test (TUG) (approximately 10-ft) [9] that has been used to determine fall-risk among older adults [7]. Previous literature indicated that the shorter distance is more feasible for use in a home setting, and has been used to differentiate between physical independence and dependence, as well as to identify fallers among older adults [7, 22]. Moreover, the 8-ft up-and-go test has previously been identified as a critical predictor of other health outcomes, such as cognitive impairment [23], functional disability [24] and mortality [21]. Based on these results, we suggest that the 8-ft up-and-go should be used in preference to other high-cost physical functional measures, since it is widely available, easy to use, and has been demonstrated to be associated with the risk of falling and other negative outcomes among older people.

Moreover, our findings show that older adults who performed lowest in the chair sit-and-reach test (lower than 1 cm) have a fall risk 1.68 times greater than those who can reach over 1 cm. These results are similar to evidence from previous studies [25, 26]. Flexibility is a crucial factor in preventing falls [25] and frailty [26] among older people. Systematic reviews have indicated that the chair sit-and-reach test is a useful alternative for testing hamstring extensibility, particular among older adults [27]. A study by Johnson et al. [28] suggests that 5 weeks of flexibility training leading to improved sit-and-reach scores can be an effective low-level exercise for improving functional fitness outcomes in community-dwelling older adults. The better sit-and-reach performance was related to other indices of improved fall risk, such as 5-times sit-to-stand and TUG scores [28]. Falls and loss of autonomy are often attributed in large part to musculoskeletal impairments in older adults. Age-related declines in flexibility contribute to the deterioration of bones and the skeletal muscles in older adults [28]. Improving lower-extremity flexibility might support musculoskeletal health, promote autonomy, and decrease fall risk in community-dwelling older adults.

The mechanisms by which physical fitness promotes increased physical health and reduces fall-risk are diverse and complex [29]. One possible explanation is that physiological reserve capacity in strength and aerobic capacity have independent effects on fall-risk [30]. This argument regards a fall as a “stress”, and takes the view that response depends upon physiological reserves not used during daily activities or exercise in the reduction of fall risk [30]. Conserving an independent lifestyle and functional mobility in later life depends to a large degree on how well maintain functional fitness performance in older adults such as dynamic balance, flexibility, agility, muscular strength, and aerobic endurance is maintained [8, 25]. Therefore, the multicomponents of functional fitness testing are crucial for evaluation of how efficiently older adults can perform the activities of daily living while reducing fall-risk.

There were several limitations in the present study. The cross-sectional design could limit causal inferences regarding the relationship between physical function and fall-risk. In the future, the prospective study will be necessary to confirm our results to predict with the risk of falling, based on the functional fitness threshold, such as the value of OR (95% CI), AUC, PPV, NPV, sensibility and specificity. Although this study validates and derives precise cut-offs, these should be subject to testing with a larger representative national population of older people. Moreover, a direct comparison of functional fitness tests measured by different fall measurements may be appropriate. In addition, the number of male participants was low (22%). A well-designed, large-scale representative sample is needed to assess the physical functions associated with fall-risk in older men. Although, this study was based on community-dwelling older adults and had an excellent response rate, it inescapably suffers from the limitations of sample representativeness. For instance, these cut-off points apply to older adults with similar characteristics to those in our study. The participants in this study were relatively healthy, so caution is required in generalizing from them, even for other sections of the older population. However, this study did not control for cognitive function as a confounder, which is known to decrease with age [31] and has also been negatively associated with physical function [32]. Lastly, the fall-risk questionnaire was self-reported and could be subject to bias. Other important covariates which might affect fall-risk, such as medical history, the circumstances of the fall, daily physical activity [33], uptake of medications and home environment, must be considered.

## Conclusions

Functional fitness capacity is an essential factor for predicting fall-risk among older adults. There is a linear dose-response association between each functional fitness index and high fall-risk. Our findings highlight the potential for tailored interventions to reduce fall-risk according to the needs of older adults with different physical functional deficiencies. Older adults who score 20 times or more in the 30-s arm-curl test and have a hand-grip strength of 25.4 kg may be at less risk of falling. Those who score less than 20 times for the 30-s sit-to-stand test might have a higher fall-risk. Our findings suggest that older people who score below 10 s for the single-leg stance test and over 6.6 s for the 8-ft up-and-go test may be at greater risk of falling. It is also strongly recommended that a chair sit-and-reach test involving over 1 cm of hip outstretch might reduce fall risk. In addition, the steps of less than 92 for the 2-min step test should be classified as indicating low cardiorespiratory fitness and might identify a primary targeting group at risk of falling. Overall, these cut-off points can be used by the community working towards fall prevention as a way of establishing a starting point for designing an effective intervention. Further researches targeting larger or more representative older populations are justified to confirm these findings.

## Supplementary Information

**Additional file 1.**

## Data Availability

All data generated or analyzed during this study are included in this published article.

## Abbreviations

BMI: Body mass index
FRQ: Fall Risk Questionnaire
SD: Standard deviation
ORs: Odds ratios
CI: 95% confidence intervals
β: Beta values
