# Supplementary material for: Is functional fitness performance a useful predictor of risk of falls among community-dwelling older adults?
Source: Arch Public Health. 2021 Jun 18;79:108. doi: 10.1186/s13690-021-00608-1 (PMC8212534; doi:10.1186/s13690-021-00608-1)
Supplement: Supplementary file 1 — Additional file 1. [file 13690_2021_608_MOESM1_ESM.docx]

Additional Table 1

| **Variable** | **Fall-risk score (continuous)** | | |
| --- | --- | --- | --- |
|  | **β (95% CI)** | ***p*-value** | **R^2^** |
| Sit-to-stand | .01 (-.08, -.11) | .78 | **0.18** |
| Arm curl | -.05 (-.14, .03) | .24 |  |
| Single-leg stance | -.09 (-.18, -.01) | **.03*** |  |
| 8-foot up-and-go | .28 (.18, .38) | **<.001*** |  |
| Chair sit-and-reach | -.07 (-.15, .00) | .06 |  |
| 2-minute step | .02 (-.07, .10) | .73 |  |
| Hand Grip Strength | -.11 (-.19, -.03) | **.005*** |  |

Abbreviations: β (95% CI) = standardized regression coefficients and 95% confidence intervals.

Adjusted for age, gender, and Body Mass Index (BMI).
